# Complementary Feeding Practices in a Cohort of Italian Late Preterm Infants

**DOI:** 10.3390/nu10121861

**Published:** 2018-12-02

**Authors:** Maria L. Giannì, Elena Bezze, Lorenzo Colombo, Camilla Rossetti, Nicola Pesenti, Paola Roggero, Patrizio Sannino, Salvatore Muscolo, Laura Plevani, Fabio Mosca

**Affiliations:** 1 Neonatal Intensive Care Unit, Fondazione IRCCS Cà Granda Ospedale Maggiore Policlinico, via Commenda 12, 20122 Milan, Italy; elena.bezze@policlinico.mi.it (E.B.); lorenzo.colombo@policlinico.mi.it (L.C.); cami.brown95@gmail.com (C.R.); nicola.pesenti@hotmail.it (N.P.); paola.roggero@unimi.it (P.R.); salvatore.muscolo@policlinico.mi.it (S.M.); laura.plevani@policlinico.mi.it (L.P.); fabio.mosca@unimi.it (F.M.); 2 Department of Clinical Sciences and Community Health, University of Milan, Via San Barnaba 8, 20122 Milan, Italy; 3 S.I.T.R.A. Basic Education Sector, Fondazione IRCCS Cà Granda Ospedale Maggiore Policlinico, Via Francesco Sforza 28, 20122 Milan, Italy; patrizio.sannino@policlinico.mi.it

**Keywords:** late preterm infants, complementary feeding, complementary foods

## Abstract

Limited data are available on complementary feeding in preterm infants, who show increased nutritional needs and are at risk of altered postnatal growth. The aim of this study was to investigate the timing and content of complementary feeding in a cohort of late preterm infants. We conducted a prospective, observational study, including mothers who had given birth to infants admitted to level I or II of care with a gestational age between 34 and 36 weeks. Mothers were contacted at 3, 6 and 12 months after delivery by phone calls and were asked about their infant’s mode of feeding and the timing and schedule of the introduction of different solid foods types. A total of 49 mothers and 57 infants completed the study. The mean postnatal age of the introduction of complementary foods was 5.7 ± 0.7 months. Low energy and/or low protein-dense foods were first introduced in most infants. Fruit as the first type of complementary food in the infant’s diet was associated with a 1.6-month advance in initiating complementary feeding. The present findings provide further insight into complementary feeding practices in late preterm infants and underline the need for specific recommendations addressing this vulnerable population.

## 1. Introduction

The introduction of complementary feeding, that is, feeding of food aside from milk in the infant’s diet, together with a reduction of the milk intake [1], puts the infant at risk of nutritional deficits or excesses, since it occurs during a period characterized by rapid growth and development [2,3]. In addition, extensive evidence indicates that inadequate nutrition during early life may potentially have negative long-term health effects [4]. Hence, the timing of the introduction of complementary foods and its related adequateness and safety are critical to meeting infants’ nutritional requirements and promoting their good future health [5]. Moreover, the complementary period allows the infant to be exposed to new foods and feeding experiences, which contribute to modulation of the brain connections implicated in the mechanisms controlling for food intake, thus affecting long-term eating behavior [2]. The introduction of complementary foods also gradually guides the infant towards eating family foods and attaining the family’s diet model [3,6].

Despite the importance of the complementary period from a nutritional and developmental perspective, limited attention has been paid to it, whereas a large amount of literature on the period of milk feeding is available [7]. According to international literature, the recommended timing for introducing complementary feeding ranges from four to six months [1,3]. However, practices in complementary foods vary greatly between and within countries. Even more limited data are available on the optimal age and type of foods to be introduced in preterm infants, who have increased nutritional needs and are at higher risk of postnatal growth retardation [8].

Nearly three-quarters of preterm births are late preterm infants, defined as infants born between 34 0/7 and 36 6/7 weeks of gestation [9]. In addition to showing an increased risk of morbidity and mortality, late preterm infants have feeding difficulties, including avoidance behavior, and are at increased risk of an altered growth pattern, both in terms of growth quantity and quality [10,11,12].

The aim of the present study was to investigate the timing and content of complementary feeding in a cohort of late preterm infants.

## 2. Materials and Methods

### 2.1. Design and Setting

We conducted a prospective, observational study that included mothers who had given birth to late preterm infants admitted to the authors’ institution from May to July 2016.

The hospital covers almost 6000 pregnancies per year. As previously reported [13], late preterm infants with a birth weight ≥1900 g, irrespective of gestational age, are admitted to level I of care (well newborn nursery), provided that clinical conditions are stable, and no nutritional support is needed. Infants with birth weight <1900 g and/or infants requiring any type of ventilatory and/or cardiovascular and/or nutritional support, are admitted/transferred to either level II (special care nursery) or III (neonatal intensive care) of care, according to their clinical conditions.

Approval from the institutional review board was granted, and written informed consent was obtained from the mothers.

### 2.2. Sample

The inclusion criteria were as follows: Mothers with good comprehension of the Italian language, who gave birth to newborns with a gestational age from 34 0/7 to 36 6/7 weeks.

The exclusion criteria were mothers who gave birth to newborns admitted to level III care and/or with congenital and/or chromosomal diseases and/or brain, metabolic, cardiac or gastrointestinal diseases.

### 2.3. Data Collection Procedures

Mothers were enrolled on the day that their infant was discharged. At enrolment, the following maternal variables were collected through a face-to-face interview: Mode of delivery (vaginal delivery/caesarean section), parity, singleton or twin pregnancy, maternal education (classified as low (≤13 years) or high (>13 years), ethnicity (Caucasian, Hispanic American, Asian) and age. The following neonatal variables were also recorded: Sex, birth weight, gestational age, length of hospital stay, Apgar score that assesses newborn’s adaptation to extrauterine life through 5 items (skin color, heart rate, reflexes, muscle tone, respiration) at 5 min, and mode of feeding at discharge. Corrected age was calculated from the chronologic age, adjusting for gestational age.

Mothers were contacted at 3 (±7 days), 6 (±7 days) and 12 (±14 days) months after delivery by phone calls by two investigators. Following a structured interview, mothers were asked whether their infant had been breastfed and/or had received any non-breastmilk foods or nutritive liquids during the last 24 hours. Mode of breastfeeding was then categorized according to the World Health Organization [14]. Specifically, infants were categorized as exclusively breastfed when they had received only breast milk and no other food or drink, not even water, with the exception of oral rehydration solutions, or drops/syrups of vitamins, minerals or medicines. Infants were categorized as predominantly breastfed when they had been fed breast milk as a predominant source of nourishment, but they had also received liquids, such as water and water-based drinks or fruit juice. Partial breastfeeding included infants that were fed both with breastmilk and formula. Exclusively formula feeding indicated infants being fed with only formula.

If the mothers reported that their infant had been fed a semi solid and/or a solid food, they were then asked about the timing of the first introduction of complementary foods; they were asked about the timing and schedule of the introduction and about which food type was the first to be introduced in the infant’s diet. According to Carletti et al. [15], the food types were grouped in the following categories: Fruit, vegetables (with a distinct category for tomatoes, due to their potential allergenicity), cereals (with and without gluten), meat, cured meats, legumes, dairy products, fish, eggs, industrial fruit juice, tubers, honey, sugar (added to drinks or food by the mothers), salt (added to food), cow’s milk, nuts and seeds. At the 12-month phone interview, mothers were asked when and which food types had already been introduced into the infant’s diet. In the event that a specific category of food had not been introduced into the infant’s diet, the reason for not introducing it was recorded.

### 2.4. Statistical Analysis

The data are expressed as the mean ± standard deviation or the number and percentage of observations.

For analysis, maternal age, birth weight and gestational age were redefined in two categories on the basis of their median value.

Timing of complementary food introduction was compared among groups using the *t*-test.

Associations between timing of complementary food introduction and infant (singleton vs. twins, birth weight, gestational age at birth, mode of feeding at three months) and maternal (maternal age, ethnicity, maternal education, parity) variables, and type of first complementary food introduced (fruit vs. others) were first assessed using univariate linear regression analysis. We decided to categorize this latter variable as fruit vs. others on the basis of the results reported by Carletti et al. [15], who found that, in their cohort of infants, fruit was the first complementary food type to be introduced.

A multivariate linear regression analysis was further performed in order to investigate the variables that were independently associated with the timing of complementary foods introduction. When adjusting the model, we included the maternal and infant characteristics that showed a significant association with the timing of complementary foods introduction at univariate analysis and/or, although not showing a significant association at univariate analysis, were considered important from a clinical point of view with regard to the timing of complementary foods introduction and/or, according to literature, could have affected the timing of complementary foods introduction.

## 3. Results

Of the 146 late preterm infants that were born during the study period, 126 were eligible for the study. A total of 53 mothers and 64 infants were enrolled. However, 49 mothers and 57 infants completed the study. The basic characteristics of the mother-infant pairs that have completed the study are shown in Table 1. No difference was found between the basic characteristics of the mother-infant pairs that have completed the study and those of the mother-infant pairs that have not completed it.

At discharge, infants were exclusively breastfed and partially breastfed respectively in 34% and 66% of cases. At three months the percentage of exclusively breastfed infants dropped to 24% whereas predominant breastfeeding, partial breastfeeding and exclusively formula feeding was registered in 9%, 30% and 37% of infants, respectively. At six months only 3.5% of infants were still exclusively breastfed, 2% were predominantly breastfed, 3.5% were partially breastfed and 10% were exclusively formula fed. A total of 26% of the infants initiated complementary feeding while being breastfed whereas complementary feeding was introduced in 14% and 41% of infants during partial breastfeeding and formula feeding, respectively. At 12 months of age, 22% of the enrolled infants were still breastfed.

The mean postnatal age of the introduction of complementary foods was 5.7 ± 0.7 months. Specifically, 27 mothers began weaning at the completion of six months of age. When considering the corrected age, the mean age at the introduction of complementary foods was 4.6 ± 0.7 months.

In Table 2 the mean postnatal age of the introduction of complementary foods according to maternal and infant characteristics and type of complementary foods first introduced is reported. No statistical difference according to the investigated characteristics was found except with regard to the type of complementary food first introduced.

Mothers initiated complementary feeding following the advice of their pediatrician in 88% of cases but decided on their own in 12% of cases. In 94% of cases, mothers followed the advice of their pediatrician for the subsequent timing of complementary food introductions. Vegetable soup with cereals and vegetable soup with cereals and meat were the types of complementary food first introduced by 33% and 31% of mothers, respectively. Fruit, vegetable soup with meat, and vegetable soup were first introduced by 16%, 14%, and 6% of mothers at 4.5 ± 53, 5.3 ± 1.0, 5.5 ± 0.5 months of infant’s age, respectively.

At univariate analysis, no significant association was found between the timing of the introduction of complementary feeding with any of the maternal and infant characteristics. On the contrary, the introduction of fruit as the first type of complementary food in the infant’s diet resulted to be associated with a 1.4-month advance in initiating complementary feeding introduction (Table 3).

The tested multivariate linear regression model resulted to be overall significant (*p* < 0.0001) and explained 57% of the variation in the timing of complementary feeding initiation (*R*^2^ = 0.57; adjusted *R*^2^ = 0.49). Specifically, no significant association was found between the timing of the introduction of complementary feeding with any of the maternal and infant characteristics. The introduction of fruit as the first type of complementary food in the infant’s diet was associated with a 1.6-month advance in initiating complementary feeding (Table 4).

The percentage of infants that have been introduced different types of complementary foods by the completion of 12 months of age and their mean postnatal age of introduction for different food types are shown in Table 5.

A total of six categories of foods (fruit, vegetables, tubers, cereal with and without gluten, white and red meat, dairy products) were introduced in all infants within the first year of life.

When fish was not introduced, the reason stated by all mothers was the fear of the occurrence of an allergic reaction. The majority of infants were introduced cured meats before completion of the first year of life whereas industrial fruit juice was introduced in the infants’ diet in nearly one-third of cases. Cow’s milk and salt were introduced in a quarter of cases, and honey in 16% of cases.

The reasons most frequently reported for not introducing these food types was either medical advice or maternal choice. In regard to eggs, the most frequent reasons reported for not introducing this type of food were mostly related to medical advice and maternal choice, whereas the fear or occurrence of an allergic reaction was indicated only in 8% of cases. Similarly, in regard to tomatoes, medical advice or maternal choice was reported by 32% and 42% of mothers, respectively, whereas fear or occurrence of allergic reaction was reported only by 17%. Not applying to the infant’s family diet, maternal choice and infant taste were equally reported as reasons for not introducing legumes before the completion of one year of age. Reasons for not introducing nuts and seeds were different, and they mostly included medical advice, maternal choice, fear or occurrence of allergic reactions and fear for suffocation (Table 6). Sugar was introduced in just one case before 12 months of age.

## 4. Discussion

The results of the present study indicate that complementary feeding in this cohort of late preterm infants was started within the fourth and the sixth month of postnatal age, following pediatrician’s advice in most cases. With regard to the content of complementary foods, low energy and/or low protein-dense foods, such as vegetable soup with or without cereals or fruit, were first introduced in the diet of more than half of the infants. Moreover, specific food categories were introduced into the infants’ diet either before or after the recommended optimal time frame for their introduction.

Timing of complementary food introduction in the present study resulted to be in line with international recommendations for full-term infants [3]. However, it must be considered that only a limited percentage of mothers met the World Health Organization recommendations, that advocate exclusive breastfeeding for six months and the introduction of complementary foods not before the completion of six months of life. Accordingly, in the present study, a lower rate than desired for exclusive breastfeeding at six months in this population was found [14,16]. Consistent with our findings, Carletti et al. [15] investigated complementary feeding practices in a cohort of 400 Italian infants with a birth weight ≥2000 g and a gestational age ≥36 weeks. The authors reported partial compliance with the World Health Organization recommendations in most cases [14]. Nevertheless, regarding the mode of feeding before the initiation of complementary feeding, the rates of breastfeeding in the present study were consistent with previous data available in the literature [17,18].

When considering the corrected age, the timing of the introduction of complementary foods in the present study was 4.6 ± 0.7 months. Data reporting the timing of complementary food introductions in preterm infants differ greatly in the literature [19,20,21,22]. The importance of considering the developmental milestones of each infant when introducing complementary foods, in addition to their nutritional status, has been underlined by Palmer et al. [19]. Accordingly, the authors have indicated three months of corrected age as appropriate timing from a developmental point of view for most infants in developed countries [19]. In contrast, Gupta et al. [20] conducted a randomized controlled trial in lower middle-income countries that aimed to evaluate the effect of initiating complementary feeding at four versus six months of corrected age in infants born at a gestational age less than 34 weeks. The authors found that the rate of hospitalization in infants that had begun complementary feeding at four months was higher. In light of these findings they concluded that six months of corrected age appears to be more appropriate timing for initiating complementary feeding in preterm infants than four months of age. These findings are in contrast to those reported by Marriot et al., who randomized preterm infants to receiving complementary foods either at 6.3 weeks of corrected age or at 9.9 weeks of corrected age [21]. The authors reported that earlier complementary food introductions were associated with better growth and higher serum and haemoglobin levels than later introductions [21]. However, it must be considered that the study by Gupta et al. [20] was conducted in developing countries. Hence, the related findings may not be applicable to different environmental contexts, such as those of developed countries. Yrjänä et al. [22] investigated the timing of complementary foods in a cohort of 664 preterm infants. According to the results of this latter study, late preterm infants initiated complementary feeding at a median corrected age of 1.9 months, without having an increase in the incidence of food allergies or atopic dermatitis.

In the present study, we did not find any significant association between maternal characteristics or the mode of breastfeeding at three months and the timing of initiating complementary feeding. Contrary to our results, a positive association between low socioeconomic state, low maternal education, maternal age <30 years, never being breastfed and earlier complementary food introductions [23,24,25] has been reported. It can be speculated that the lack of association between maternal age and earlier complementary feeding introduction in the present study could be partially, due to the fact that, in our cohort, only four of the enrolled mothers were younger than 30 years. Moreover, the finding that a low socioeconomic state and a low maternal education was not associated with an advance in the timing of complementary feeding introduction is actually consistent with the finding that most mothers followed their pediatrician’s advice. It may also reflect the Italian health care system organization that offers primary care service at a community level to all pediatric patients.

With regard to the association between the degree of prematurity and timing of complementary food introduction, data reported in the literature are not consistent [25,26].

According to the present findings, infants receiving fruit as their first complementary food resulted in complementary feeding starting 1.6 months before the infants receiving first any other type of complementary food. This finding could be partially explained by the fact that the early introduction of fruit as food, aside from milk, could be regarded in some cases, both by parents and pediatricians, as a way of testing the potential acceptance and competency of being spoon-fed rather than being considered as the actual complementary feeding initiation. The reason for choosing fruit, rather than another type of food for this scope, may reflect the Italian socio-cultural environment, which has been historically characterized by the preferential use of fruit as the first type of food for complementary food introduction [15,27]. Moreover, low energy and/or low protein-dense foods, such as vegetable soup with or without cereals and fruit were first introduced in more than half of the infants. Consistent with these results, available data in the literature indicate that complementary food introductions in preterm infants are usually carried out with low energy and/or low protein-dense foods [26,28,29]. Nonetheless, starting complementary feeding with high energy and/or high protein-dense foods might be desirable in order to meet the high nutritional requirements of these vulnerable infants and prevent the risk of growth faltering and increased adiposity [12,30].

In the present study, the timing of introduction of specific food categories into the late preterm infants’ diet appears to be similar to that of full-term infants [15]. Consistently with our results, Carletti et al. [15] reported that fruit was the first food to be introduced in the diet, followed by vegetables, cereals, milk products and meat whereas eggs and legumes had not been introduced in 53% and 74% of infants, respectively, by the age of 12 months.

Remarkably, in the present study, most mothers followed their pediatrician’s advice on the content and initial timing of complementary feeding. This finding suggests that health care professionals are regarded as a credible source of information. However, the findings of the present study further highlight great disparities in complementary feeding practices, which reflect the lack of specific recommendations addressing late preterm infants. Moreover, the present findings indicate that pediatricians do not always strictly adhere to the available recommendations for full-term infants with regard to the introduction of specific foods categories [3]. Accordingly, potentially allergenic foods, such as tomatoes, nuts and seeds, were not introduced into the infant’s diet (except for in a limited number of cases), due to fear of allergic reactions. Consistently with these results, Carletti et al. [15] found that in their cohort of infants the introduction of tomatoes, egg, fish, nuts and seeds was delayed. Furthermore, 26%, 32% and 16% of mothers introduced salt, fruit juice and honey in the infant’s diet, respectively, even though their introduction before the completion of 12 months of age should be avoided [3]. Cow’s milk was introduced in the infant’s diet in 26% of cases before the end of the first year of age, although its consumption has been associated with excessive early protein intake, which is a well-known risk factor for an increased risk of obesity later in life [31].

In light of the strict interrelationship between early nutrition and long-term health, these findings highlight the need for specific recommendations on the optimal timing and strategy of complementary food introduction in late preterm infants, with a special focus on their specific nutritional requirements.

The main strength of this work is the prospective, longitudinal design. This study has some limitations, since the data concerning the timing and content of complementary feeding refer to a cohort of Italian infants and may not apply to the late preterm infants born in other developed countries. Moreover, no data on infants’ growth pattern after discharge and the occurrence of feeding difficulties have been collected. Lastly, the statistical power of the study is limited, due to the relatively small number of enrolled infants.

## 5. Conclusions

The results of the present study provide further insight into complementary feeding practices in late preterm infants. Timing of complementary foods introduction in this population appears to be in line with recommendations for full-term infants. However, low energy and/or low protein-dense foods are first introduced in more than half of the infants despite their high nutritional requirements and their risk of altered growth and body composition. Moreover, specific food categories are introduced into the infant’s diet either before or after the recommended optimal time frame.

In view of the importance of the complementary feeding period, in terms of promoting health outcomes, specific recommendations addressing late preterm infants are desirable.

## Figures and Tables

**Table 1:** Basic characteristics of the mother-infant pairs that have completed the study.

Subjects	Basic Characteristics
Mothers (*n* = 49)	Mean ± SD
Age (years)	36.3 ± 5.4
	*n* (%)
Ethnicity	
Caucasian	43 (88)
Hispanic American	5 (10)
Asian	1 (2)
Maternal education level	
≤13 years	25 (51)
>13 years	24 (49)
Caesarean section	33 (67)
Primiparous	26 (53)
Singleton pregnancy	40 (82)
Infants (*n* = 57)	Mean ± SD
Gestational age at birth (weeks)	35.2 ± 0.8
Birth weight (g)	2564 ± 465
Apgar score at 5′	9.5 ± 0.7
Length of hospital stay (days)	8.8 ± 8.0
	*n* (%)
Twins	18 (32)
Males	26 (46)

Data are expressed as mean ± standard deviation (SD) or *n* (%).

**Table 2:** Mean postnatal age at complementary foods introduction according to maternal and infant characteristics and type of complementary foods first introduced.

Characteristics	Timing of Complementary Foods Introduction
Maternal	Months of postnatal age
Education level	
≤13 years	5.74 ± 0.7
>13 years	5.75 ± 0.7
Ethnicity	
Caucasian	5.75 ± 0.7
Non Caucasian	5.66 ± 1.0
Age	
≤38 years	5.61 ± 0.9
>38 years	5.90 ± 0.4
Parity	
Primiparous	5.61 ± 0.8
Multiparous	5.90 ± 0.5
Infants	
Singleton	5.76 ± 0.7
Twins	5.66 ± 0.5
Gestational age	
≤35 weeks	5.68 ± 0.9
>35 weeks	5.80 ± 0.5
Birth weight	
≤2520 g	5.58 ± 0.7
>2520 g	5.87 ± 0.6

Data are expressed as Mean ± SD.

**Table 3:** Univariate regression analyses of the association between maternal and neonatal characteristics, mode of feeding at three months, type of first complementary food and timing of complementary feeding.

	Timing of Complementary Feeding Initiation (Months of Postnatal Age)			
	*R* ^2^	β	*p* Value	95% CI
Maternal age (≤38 years vs. >38 years)	0.04	0.29	0.12	−0.08; 0.66
Ethnicity (Caucasian vs. non-Caucasian)	0.07	−0.18	0.52	−0.76; 0.9
Maternal education (≤13 years vs. > 13 years)	0.0	−0.007	0.9	−0.18; 0.19
Parity (primiparous vs. multiparous)	0.024	0.22	0.24	−0.15; 0.60
Gestational age (≤35 weeks vs. >35 weeks)	0.004	0.08	0.65	−0.29; 0.46
Birth weight (≤2520 g vs. >2520 g)	0.04	0.29	0.11	−0.07; 0.66
Being twin vs. singleton	0.004	0.09	0.64	−0.31; 0.50
Mode of feeding at three months (exclusively breastfeeding vs. predominantly/partial breastfeeding and formula feeding)	0.02	−0.22	0.29	−0.65; 0.20
Type of first complementary food (fruit vs. others)	0.48	−1.4	<0.0001	−1.8; −1.03

*R*^2^ = Coefficient of determination; β = Unstandardized Beta coefficient; 95% CI = 95% Confidence Interval.

**Table 4:** Multivariate regression analysis of the association between maternal characteristics, infant’s characteristics, mode of feeding at three months, type of first complementary food and timing of complementary feeding.

	Timing of Complementary Feeding Initiation (Months of Postnatal Age)		
	β	*p* Value	95% CI
Maternal age (≤38 years vs. >38 years)	0.22	0.18	−0.106; 0.54
Ethnicity (Caucasian vs. non-Caucasian)	0.50	0.09	−0.08; 1.09
Maternal education (≤13 years vs. >13 years)	−0.06	0.37	−0.22; 0.08
Parity (primiparous vs. multiparous)	0.12	0.57	−0.31; 0.55
Gestational age (≤35 weeks vs. >35 weeks)	−0.26	0.12	−0.62; 0.08
Birth weight (≤2520 g vs. >2520 g)	0.04	0.85	−0.35; 0.43
Being twin vs. singleton	0.14	0.58	−0.37; 0.66
Mode of feeding at three months (exclusively breastfeeding vs. predominantly/partial breastfeeding and formula feeding)	0.13	0.52	−0.29; 0.56
Type of first complementary food (fruit vs. others)	−1.6	<0.0001	−2.12; −1.15

β = Unstandardized Beta coefficient; 95% CI = 95% Confidence Interval.

**Table 5:** Percentage of infants that have been introduced different types of complementary foods by the completion of 12 months of age and mean postnatal age (months) of food type introduction.

Type of Food	% of Infants	Postnatal Age of Introduction (Months)
Fruits	100	5.8 ± 0.8
Vegetables	100	6.02 ± 0.5
Tubers	100	6.04 ± 0.5
Cereal without gluten	100	6.08 ± 0.5
White meat	100	6.39 ± 0.7
Dairy products	100	6.5 ± 0.98
Cereal with gluten	100	7.27 ± 1.0
Fish	98	7.35 ± 0.9
Red meat	100	7.8 ± 1.2
Cured meats	91	8.18 ± 3.1
Legumes	97	8.6 ± 1.5
Eggs	75	9.95 ± 1.3
Nuts and seeds	84	10.0 ± 2.05
Tomatoes	70	10.1 ± 1.4
Industrial fruit juice	32	10.1 ± 1.5
Salt	26	11.5 ± 0.8
Cow milk	26	11.5 ± 0.7
Honey	16	11.6 ± 1.06

Data are expressed as mean ± standard deviation (SD).

**Table 6:** Reasons (%) stated by mothers for not introducing some food types in their infant’s diet.

	Reasons for Not Introducing Some Food Types in the Infant’s Diet (%)					
	Medical Indication	Maternal Choice	Fear or Occurrence of Allergic Reactions	Infant Does Not Like the Food	It Does Not Apply to the Family Diet	Fear of Suffocation
Fish	-	-	100	-	-	-
Cured meats	25	75	-	-	-	-
Legumes	-	34	-	33	33	-
Eggs	34	33	8	8	17	-
Nuts and seeds	18	32	18	-	3	29
Tomatoes	33	42	17	8	-	-
Industrial fruit juice	45	49	-	3	3	-
Salt	88	12	-	-	-	-
Cow milk	45	48	-	7	-	-
Honey	85	15	-	-	-	-

Data are expressed as %.
